# Hepatotoxicity: Molecular Mechanisms and Pathophysiology

**DOI:** 10.3390/ijms20010211

**Published:** 2019-01-08

**Authors:** Rolf Teschke

**Affiliations:** Department of Internal Medicine II, Division of Gastroenterology and Hepatology, Klinikum Hanau, D-63450 Hanau; Academic Teaching Hospital of the Medical Faculty, Goethe University Frankfurt/Main, Frankfurt am Main, Germany; rolf.teschke@gmx.de; Tel.: + 49-8-6121-21859

The current Special Issue is devoted to the broad spectrum of hepatotoxicity with its molecular mechanisms and pathophysiology, presented in eight publications [1,2,3,4,5,6,7,8]. Scientists were from various countries, including the US, Mexico, the Czech Republic, Germany, Portugal, China, and Japan. Contributions considered various types of experimental and human liver injury, elicited by a number of causal conditions and substances.

In the first article of this special issue, Sena et al. [1] from Portugal used a most interesting animal model of fatty liver caused by a high-fat diet (HFD). Such a kind of diet is innovative because it also triggers diabetes, and therefore, provides an excellent approach mimicking non-alcoholic fatty liver disease (NAFLD) in humans. This disorder is clinically most frequently observed in patients with overweight or morbid obesity associated with diabetes and the metabolic syndrome. The authors described an attenuation of hepatic steatosis by the use of α-lipoic acid (α-LA), which is a drug for the treatment of a variety of human diseases due to its protective effects injury caused by reactive oxygen species (ROS). In their study, Sena et al. confirmed that α-lipoic acid reduces oxidative stress and expanded their experiments to elucidate the mechanisms of the protective effect. They finally attributed the positive effects to the action of nuclear factor E2 (erythroid-derived 2)-related factor-2 (Nrf2). Of interest is the use of Goto Kakizaki (GK) rats, which typically exhibit an inferior body weight compared to age-matched Wistar rats. Feeding a HFD for three months to GK rats significantly increased body weight compared to control GK rats. Livers from the GK rats fed with a HFD were significantly greater in size compared to control GK rats and significantly smaller in the α-LA-treated GK rats compared to the GK rats fed with a HFD. It remains unclear and was not studied whether the used animal model causes steatosis by microsomal oxidative stress or possibly mitochondrial oxidative stress. However, such specificities are difficult to assess and to differentiate from each other.

For the second most interesting contribution, Zárybnický et al. [2] from the Czech Republic used human liver slices derived from five livers to study acute liver injury caused by the monoterpenes R-Pulegone (PUL) and R-Menthofuran (MF), abundantly present in plants of the Lamiaceae family. Pulegone and MF are also major constituents of several mint (*Mentha*) species and their derived volatile oils, including peppermint (*M. piperita*), spearmint (*M. spicata*), European pennyroyal (*M. pulegium*), and American pennyroyal (*H. pulegioides*). They are used for flavoring of foods and drinks, in herbal medicinal products, and cosmetics. Pulegone was determined to be the major constituent of pennyroyal oil and MF as one of the major metabolites of PUL in the body. Although their hepatotoxicity was shown in rodent species, information on their injurious effects in humans has been limited. Using in other sets also acetaminophen (known also as paracetamol) as a control substance, the toxicity of these three substances showed significant inter-individual variability of their toxic effects. Pulegone was toxic in all liver samples, whereas MF and acetaminophen only in two and three liver samples, respectively. Marked inter-individual variabilities in all our results demonstrated the high probability of significant differences in the hepatotoxicity of tested compounds among individuals. Pulegone also changed miRNA expression more significantly than MF and acetaminophen. The most pronounced effect was a marked decrease of miR-155-5p expression caused by PUL even in non-toxic concentrations in all five liver samples, suggesting that miR-155-5p could be a good diagnostic biomarker of early PUL hepatotoxicity. Most importantly, PUL, MF, and acetaminophen share the common feature causing intrinsic liver injury in humans and experimental animals. Therefore, having used human liver slices was an ideal approach and should be used more often in studies on intrinsic drug-induced liver injury (DILI) and herb-induced liver injury (HILI). 

The publication by Hirasawa et al. [3] from Japan focused on important clinical issues related to nevirapine, a potent non-nucleoside reverse transcriptase inhibitor (NNRTI) drug used in combination with nucleoside analogues for treatment of human immunodeficiency (HIV). Nevirapine is commonly well tolerated but may cause potentially severe rash and occasionally severe or even fatal hepatic hypersensitivity in the early weeks of treatment. The authors described its interaction with human leucocyte antigen (HLA), the Peptide Binding Groove of HLA-DRB1*01:01, and its effect on the conformation of HLA–Peptide Complex. Human leucocyte antigen-DRB1*01:01 has been shown to be involved in nevirapine-induced hepatic hypersensitivity reactions. Additionally, in silico docking simulations and molecular dynamics simulations were performed to predict the interaction mode of nevirapine with the Peptide Binding Groove of HLA-DRB1*01:01 and its possible effect on the position and orientation of the ligand peptide derived from hemagglutinin (HA). In silico analyses suggested that nevirapine interacts with HLA-DRB1*01:01 around the P4 pocket within the peptide binding groove and the HA peptide stably binds on top of nevirapine at the groove. The analyses also showed that binding of nevirapine at the groove will significantly change the inter-helical distances of the groove. An in vitro competitive assay showed that nevirapine increases the binding of the HA peptide to HLA-DRB1*01:01 in an allele-specific manner. These results indicate that nevirapine might interact directly with the P4 pocket and modifies its structure, which could change the orientation of loaded peptides and the conformation of HLA-DRB1*01:01; these changes could be distinctively recognized by T-cell receptors. Through this molecular mechanism, nevirapine might stimulate the immune system, resulting in hepatic hypersensitivity reactions, but their diagnostic relevance remains to be clarified.

The authors Zhang et al. [4] from Hong Kong (China) studied clinically important molecular mechanisms involved in herb-induced liver injury (HILI) by traditional Chinese medicine (TCM) due to oxidative stress. Another focus was on experimental evidence-based literature review and network pharmacology study. They pointed out in detail that clinicians had often used Roussel Uclaf Causality Assessment Method (RUCAM) for causality assessment in HILI cases because this method would provide a high degree of certainty. The RUCAM was deemed as a validated approach to assigning key points for liver-specific clinical symptoms in HILI cases. Zhang et al. also referenced the updated version of RUCAM and discussed results obtained from updated RUCAM-based case reports containing TCM administration alluding to a prospective and large-scale study of 21,470 patients without liver diseases, who had been treated with 11 different herbal TCMs, including *Bupleuri radix*, *Scutellariae radix*, and *Glycyrrhizae radix*. In essence, 26 patients (0.12%) experienced high values of alanine aminotransferase (ALT): ≥5 x the upper limit of normal. Associated RUCAM-based causality grades for TCM-treated patients were probable in 8/26 patients, possible in 16/26, and excluded in 2/26. Therefore, 24 patients (0.11%) might have experienced TCM-induced liver injury, and the most suspicious TCMs with hepatotoxic effects in this study were *Bupleuri radix* and *Scutellariae radix*, suggesting that these two herbs are mainly prone to induce liver injury. The authors also suggest additional focus on the herbal treatment of greater celandine and kava, since several RUCAM-based causality assessments indicated that liver injury could be generated by these herbs. Finally, the authors have conducted a literature search targeting hepatotoxic TCM with RUCAM-based high causality grading, showing that *Polygonum multiflorum* was the highly probable TCM in 65 out of 114 cases.

In their stimulating report, Mendez-Sanchez et al. [5] from Mexico discussed new mechanistic aspects in non-alcoholic steatohepatitis (NASH). The authors regret that the cascade of events underlying the progression of non-alcoholic fatty liver disease (NAFLD) to NASH and then cirrhosis is largely unknown and not based on a single theoretical concept, although myriads of factors have been reported to be associated with NASH progression. Among the proposed factors are insulin resistance, inflammation, oxidative stress, and bile acid toxicity. However, new conceptual approaches focus more on the adipose tissue (AT) and its modulating lipotoxic property on liver function. In more detail, the release of fatty acids from dysfunctional and insulin-resistant adipocytes results in hepatic lipotoxicity. This is caused by the accumulation of triglyceride-derived toxic metabolites and the subsequent activation of inflammatory pathways, cellular dysfunction, and lipoapoptosis. Preferentially visceral AT comprises multiple cell populations that produce adipokines and insulin-like growth factor, plus macrophages and other immune cells that stimulate the development of lipotoxic liver disease. These biomolecules have recently been linked with many digestive diseases and gastrointestinal malignancies such as hepatocellular carcinoma. Evidence is now accumulating that lipotoxic events modulate the natural history of liver fibrosis, substantiating a close relationship between dysregulated AT and NASH as a target organ of lipotoxicity. A good comprehension of the pathways that are related to dysregulated AT, metabolic dysfunction, and hepatic lipotoxicity could result in the development of prevention strategies and promising therapeutics for patients with NASH and subsequent cirrhosis. In essence, a closer view on adipose tissue with its modulating features on NASH is warranted. 

The study of Natarajan and Ibdah [6] from the US is devoted to the clinically important acute fatty liver of pregnancy (AFLP) and the role of 3-hydroxy fatty acid-induced hepatic lipotoxicity. Based on clinical experience and many publications on case reports and case series, consensus exists that AFLP is a serious illness for both the mother and the unborn offspring. Disease develops in the last trimester of pregnancy with significant maternal and perinatal mortality. Acute fatty liver of pregnancy is also recognized as an obstetric and medical emergency. Maternal AFLP is highly associated with a fetal homozygous mutation (1528G>C) in the gene that encodes for mitochondrial long-chain hydroxy acyl-CoA dehydrogenase (LCHAD). The mutation in LCHAD results in the accumulation of 3-hydroxy fatty acids, such as 3-hydroxy myristic acid, 3-hydroxy palmitic acid, and 3-hydroxy dicarboxylic acid in the placenta, which are then shunted to the maternal circulation leading to the development of acute liver injury observed in patients with AFLP. The authors Natarajan and Ibdah discussed the mechanistic role of increased 3-hydroxy fatty acid in causing hepatic lipotoxicity and inducing oxidative stress, mitochondrial dysfunction, and hepatocyte lipoapoptosis. Further, they also reviewed the role of 3-hydroxy fatty acids in causing placental damage, pancreatic islet β-cell glucolipotoxicity, brain damage, and retinal epithelial cells lipoapoptosis in patients with LCHAD deficiency. In essence, AFLP can best be classified as molecular disease based on a deficiency of mitochondrial LCHAD in the fetus, leading to an accumulation of 3-hydroxy fatty acids, which in turn impair mitochondrial functions and initiate a vicious circle based on triggering continuous mitochondrial dysfunction. 

The authors Schueller et al. [7] from Germany covered in their critical and excellent contribution the challenging issue of miRNAs in the pathophysiology of liver diseases and their role as potential diagnostic biomarkers in clinical medicine. They remind us that microRNAs (miRNAs) represent a new class of highly conserved small non-coding RNAs, which are involved in the regulation of gene expression by targeting whole networks of so called “targets”. The expression of miRNAs is specifically altered in virtually all acute and chronic liver diseases. In addition, miRNAs can act as mediators of inflammatory and fibrotic processes. Schueller et al. also highlighted the potential role of circulating microRNAs in diagnosis of liver diseases and critically discussed the major drawbacks that currently prevent the use of miRNAs in clinical routine. In particular, there is yet a lack of diagnostic biomarkers that would allow for early recognition of drug-induced liver injury (DILI) or herb-induced liver injury (HILI). In clinical practice, they recommended repetitive measurements of alanine aminotransferase (ALT) or alkaline phosphatase (ALP) in connection with causality assessment using the RUCAM score. For novel miRNA-based biomarkers to be used in DILI or HILI, major issues are still to be addressed. Most importantly, there is a need that the new miRNA biomarkers are liver specific and drug specific, with results derived from patients with high causality gradings for DILI or HILI, as evaluated using RUCAM. Respective references of studies have been quoted that applied the updated RUCAM version. In essence, consensus exists that new biomarkers must be validated using liver injury cases with high causality gradings after assessment by the updated RUCAM. 

The last report of the special issue was provided by the authors Brewer and Chen [8] from the US who correctly emphasized the problem of the large numbers of pharmaceuticals and supplements that have been approved by the US Food and Drug Administration (FDA)-approved, but appreciated that pharmaceuticals are evaluated for the potential to inhibit or induce cytochrome P450 (CYP) enzymes before being marketed in the US. However, regulations for herbal supplements in the US do not require surveillance or the reporting of adverse events by the manufacturer to the FDA. Thus, the data concerning the hepatotoxicity of herbal supplements are derived from case reports and series, retrospective databases, and registries. They describe in detail that the xenobiotic receptors constitutive androstane receptor (CAR) and the pregnane X receptors (PXRs) can respond to xenobiotics by increasing the expression of a large number of genes that are involved in the metabolism of xenobiotics. However, many xenobiotics can inhibit CYP activity or lead to enzyme induction, and these modifications often lead to unwanted interactions and toxicity. They also comprehensively discuss the modulating effect of CYP on liver injury induced by herbal supplements. Their expert analysis highlighted various clinical issues of CYP related to drug–drug and herb–herb interactions that initiate herb-induced liver injury (HILI). Brewer and Cheng also correctly emphasize that predicting and evaluating the risk of liver injury by herbs require both a repository of the information reported in the clinic and a standardized causality assessment method such as the updated version of RUCAM. In their opinion, the updated RUCAM now addresses previous weaknesses and streamlines the evaluation of the liver injury cases, whereby the updated definitions of the classification items reduces the variability of the assessments and the dependence upon outside experts not involved with the case, an important point they made in agreement with other scientists. 
